# The Regulatory Network of CREB3L1 and Its Roles in Physiological and Pathological Conditions

**DOI:** 10.7150/ijms.90189

**Published:** 2024-01-01

**Authors:** Ying Zhao, Zhou Yu, Yajuan Song, Liumeizi Fan, Ting Lei, Yinbin He, Sheng Hu

**Affiliations:** 1 Department of Anesthesiology and Perioperative Medicine, Xi'an People's Hospital (Xi'an Fourth Hospital), Northwest University, Xi'an, Shaanxi Province, China.; 2 Department of Plastic Surgery, Xijing Hospital, Fourth Military Medical University, Xi'an, Shaanxi Province, China.

**Keywords:** CREB3L1, Bone Morphogenesis, Neurogenesis, Endocrine, Angiogenesis, Tumorigenesis, Fibrosis.

## Abstract

CREB3 subfamily belongs to the bZIP transcription factor family and comprises five members. Normally they are located on the endoplasmic reticulum (ER) membranes and proteolytically activated through RIP (regulated intramembrane proteolysis) on Golgi apparatus to liberate the N-terminus to serve as transcription factors. CREB3L1 acting as one of them transcriptionally regulates the expressions of target genes and exhibits distinct functions from the other members of CREB3 family in eukaryotes. Physiologically, CREB3L1 involves in the regulation of bone morphogenesis, neurogenesis, neuroendocrine, secretory cell differentiation, and angiogenesis. Pathologically, CREB3L1 implicates in the modulation of osteogenesis imperfecta, low grade fibro myxoid sarcoma (LGFMS), sclerosing epithelioid fibrosarcoma (SEF), glioma, breast cancer, thyroid cancer, and tissue fibrosis. This review summarizes the upstream and downstream regulatory network of CREB3L1 and thoroughly presents our current understanding of CREB3L1 research progress in both physiological and pathological conditions with special focus on the novel findings of CREB3L1 in cancers.

## 1. Introduction

**Figure 1:**
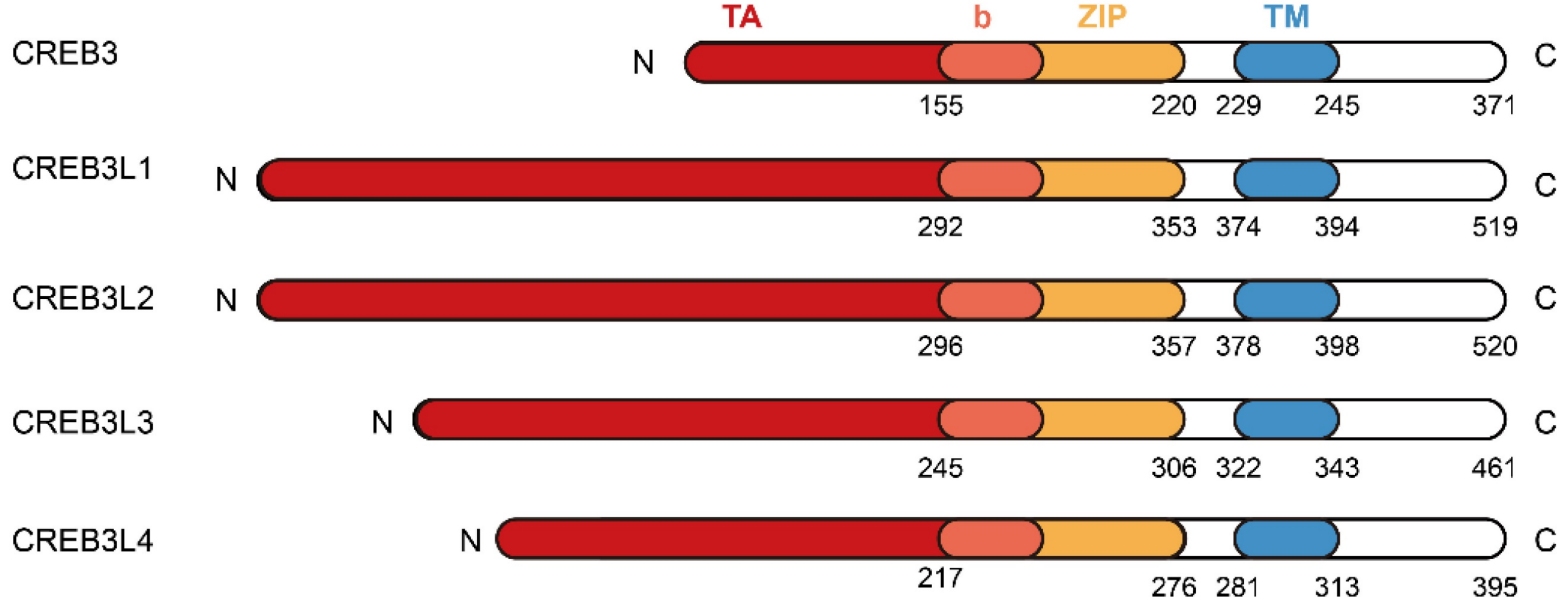
** The protein structures of CREB3 family members.** The CREB3 family comprises CREB3, CREB3L1, CREB3L2, CREB3L3, and CREB3L4. Each member of CREB3 family shares similar protein structures composed of N-terminus, transcriptional activation (TA) domain, basic leucine zipper (bZIP) domain, transmembrane (TM) domain, and C-terminus.

The cAMP responsive element (CRE) binding protein 3 (CREB3) family belongs to the bZIP transcription factors. It is composed of five members in mammals, which are known as CREB3, CREB3L1, CREB3L2, CREB3L3, and CREB3L4 1. Members of CREB3 family are highly related to Drosophila dCREB-A/BBF2 and the Activating transcription factor 6 (ATF6) 1. They have a bZIP domain to function in DNA binding and a transmembrane domain to facilitate association with the endoplasmic reticulum (ER) (Figure 1). In view of this, they act as type II membrane associated proteins with the N-terminus facing the cytoplasm and the C-terminus penetrating through the ER membrane into the ER lumen 1. In the absence of ER stress, these transcription factors are localized in ER membranes. Upon stimulation from ER stress, they are transported to the Golgi apparatus and cleaved by proteases S1P/MBTPS1 and S2P/MBTPS2 to liberate the N-terminal cytosolic domain. This process is named as regulated intramembrane proteolysis (RIP) and has been clearly delineated by professor Jin Ye 2. Then the released N-terminal domains translocate to the nuclei and serve as transcription factors to bind to the CRE sequence to regulate the transcription of target genes, thus functioning in diverse physiological and pathological conditions.

**Figure 2:**
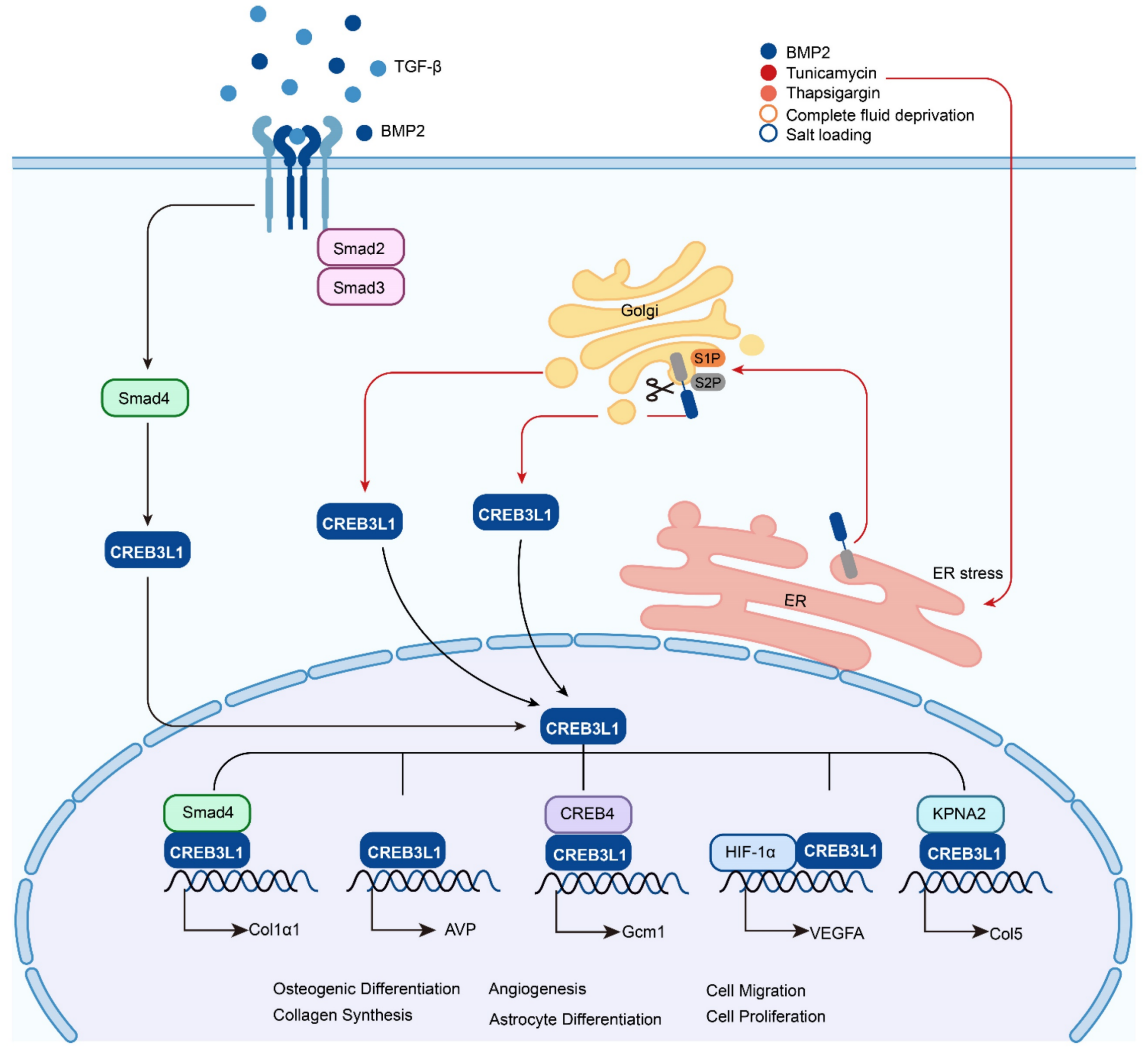
** The activation of CREB3L1 by RIP and its transcriptionally regulated downstream target genes.** Upon the chronic simulation of TGF-β, CREB3L1 can bind to Smad4 and translocate to the nuclei to activate the transcription of Col1α1, while receiving the signal of ER stress, such as treatment with BMP2, tunicamycin, thapsigargin, or suffered from fluid deprivation or salt loading, CREB3L1 on ER can be transferred to the Golgi apparatus and sequentially cleaved by S1P and S2P, then the liberated active form of CREB3L1 translocates to the nuclei to regulate the expressions of genes, for instance AVP, Gcm1, VEGFA, and Col5 by itself or interaction with other proteins.

CREB3 (also referred to as LZIP or Luman) was first identified as an interacting protein associated with herpes simplex virus VP16 associated host cellular factor (HCF) by a yeast two hybrid system, it expresses and functions in various adult and fetal tissues 4. CREB3L1 was originally named as OASIS (old astrocyte specifically induced substance) and considered to be a gene specifically expressed in the long-term cultured astrocytes 5. The human CREB3L1 gene is located at chromosome 11p11.2 and encodes a protein consisted of 519 amino acids. This protein contains an N-terminal domain and a C-terminal domain. The N-terminal domain harbors the transmembrane domain, while the C-terminal domain is within the ER lumen. In the peptide of lumenal part, CREB3L1 includes an amino acid sequence of RSLL (beginning at residue 423), which matches the R (x) x L consensus for protease S1P with the active site confronting the Golgi lumen. Under the stimulation of ER stress such as Bone morphogenetic protein 2 (BMP2) 6, tunicamycin 7, thapsigargi 7, complete fluid deprivation 8, or salt loading 8, CREB3L1 is cleaved at the above-mentioned site by S1P and then cut at the transmembrane domain by S2P 9, 10. Subsequently, the liberated N-terminal domain transfers to the nucleus and acts as a transcription factor to regulate the expressions of target genes. In addition, BMP2 and Transforming Growth Factor-β (TGF-β) can upregulate the expression of CREB3L1 and benefit the initiation of RIP to liberate the active form of CREB3L1, which then activates the transcription of genes (Figure 2). Up to now, CREB3L1 has been detected in osteoblasts of bone tissues 11, fibroblasts in skin 12, and astrocytes in the central nervous system 9, 13. Besides, CREB3L1 is also distributed in the tissues of intestine, salivary gland, and prostate 5, 14, 15.

CREB3L2 is also referred as BBF2 human homolog on chromosome 7 (BBF2H7). It locates on chromosome 7 and was identified because of the fusion of the BBF2H7 with the FUS gene on chromosome 16 in a human low grade fibromyxoid sarcoma (LGFMS) 16. CREB3L2 works as a transmembrane transcription factor with similar protein structure to CREB3L1 and is activated by RIP upon ER stress 17. CREB3L2 is expressed in the proliferating zone of cartilage, lung, spleen, gonad, and nervous system, which differs from the expression pattern of CREB3L1 obviously 17, 18. CREB3L3 is also known as CREBH and was isolated as a bZIP transcription factor specially expressed in the liver 19. Although CREB3L3 is mainly expressed in the liver, a lower expression of CREB3L3 was observed in the small intestine and stomach too 19, 20. Under ER stress condition, CREB3L3 was cleaved sequentially by S1P and S2P to generate a transcription factor. CREB3L4 was originally identified through high throughput sequencing of human cDNA libraries 21. It is also referred as AIbZIP, CREB4, or TISP40 and mainly distributes in the tissues of human prostate, brain, and pancreas. Previous study indicated that CREB3L4 appeared to function in the prostate and was involved in regulation of prostate cancer cells proliferation 22. However, there also existed study showing that CREB3L4 was specifically expressed in the mouse testis and participated in the process of spermatogenesis 23.

Generally, members of the CREB3 family mainly participate in regulating acute phase response, cell differentiation, development, protein secretion, lipid metabolism, organelle autoregulation, survival, and tumorigenesis, etc. 1, 15, 24. CREB3L1 acting as one of them exhibits distinct functions from other members. Physiologically it transcriptionally regulates the expression of various genes to modulate bone development 15, function of central nervous system 22, secretory cell differentiation 26, decidualization 27,28, and angiogenesis 29-31. Pathologically, it activates the transcriptions of downstream target genes to function in the occurrence, growth, progress, metastasis, and prognosis of tumors, such as glioma, breast cancer, and thyroid cancer. In addition, somatic mutations in CREB3L1 contribute to osteogenesis imperfecta (OI), while the generation of Fused in sarcoma (FUS)-CREB3L1 and EWS RNA Binding Protein 1 (EWSR1)-CREB3L1 lead to LGFMS and sclerosing epithelioid fibrosarcoma (SEF) respectively. Except for these mentioned above, the roles of CREB3L1 in tissue fibrosis including renal fibrosis, liver fibrosis, hypertrophic scar, and keloid are emerging. In this review we aim to thoroughly elucidate the recent findings of CREB3L1 and intend to reveal its potential roles in both physiological and pathological conditions, which may enhance the understanding of CREB3L1 and facilitate the resolution of clinical issues related to CREB3L1.

## 2. The physiological functions of CREB3L1

### 2.1. CREB3L1 promotes bone morphogenesis

CREB3L1 serving as a bone development regulator is mainly distributed in the osteoblasts of mouse osseous tissues with a high level 32. CREB3L1 deficient (Oasis-/-) mice displayed severe osteopenia characterized by diminished bone density. These attribute to the lessened expression of Type I collagen (Col1) 11. Col1 is a constituent of bone tissues, mutations in Col1 genes and defects in Col1 proteins often cause osteogenesis imperfecta (OI) 33. Col1a1 acts as a downstream target of CREB3L1, it can be transcriptionally activated by CREB3L1 via directly binding to the CRE-like nucleotide sequence present in the promoter region of Col1a1. Bone tissues from Oasis-/- mice exhibit reduced Col1 expression 11. Overexpression of CREB3L1 in osteoblast specially rescues the defects of bone formation in Oasis-/- mice through upregulating the expression of Col1 34. Interestingly, bone fracture can induce the expression of CREB3L1, but the callus density of bone tissues from OASIS-/- mice was less in contrast to the wild type. The newly formed bone in Oasis-/- mice after fracture exhibit decreased callus density, reduced bone volume, and declined callus bone strength 35. Interestingly, bisphosphonate risedronate administration benefits osteopenia treatment through attenuating the abnormal expansion of rough ER in OASIS-/- mice osteoblasts 36.

Bone morphogenetic protein 2 (BMP2) is a pluripotent factor belonging to the TGF-β superfamily. It often participates in osteogenic differentiation and bone regeneration 6. Previous study confirmed that BMP2 can upregulate the mRNA expression of CERB3L1 through transcription factor RUNX family transcription factor 2 (Runx2) and facilitate CREB3L1 cleavage to liberate the N-terminal domain in an ER stress dependent way in osteoblasts. At the same time, ER stress marker X-Box binding protein 1 (Xbp1) and the chaperone protein Heat Shock Protein Family A (Hsp70) Member 5 (HSPA5/GRP78/BiP) were induced as well 11. In human beings, compared with the preosteoblasts (including preosteoblast-S1 and preosteoblast-S2) and the intermediate osteoblasts, the active scores for CREB3L1 and RUNX2 regulons in mature osteoblasts are the highest, and the PHOSPHO1, SPNS2, and COL1A1 in the CREB3L1 regulon increase as osteoblasts mature 37. Differential proteomics-based identification of ubiquitylation substrates (DiPIUS) revealed that CREB3L1 serves as the substrate of ubiquitin ligase F-Box and WD repeat domain containing 7 (Fbxw7). Fbxw7 overexpression downregulates CREB3L1 and Col1a1 expression, whereas Fbxw7 deficiency facilitates the osteogenic differentiation of mouse mesenchymal cells, which may attribute to the accumulation of CREB3L1 38. Besides, CREB3L1 also contributes to tooth development, its mRNA expression was found in the preodontoblasts and differentiating odontoblasts of mice 39. CREB3L1 may also function in apical odontoblasts (AOds) which are responsible for tooth root formation and root regeneration and the osteogenic differentiation of periodontal ligament stem cells (PDLSCs) 40, 41.

### 2.2. CREB3L1 facilitates Neurogenesis

CREB3L1 was primarily identified as a transcription factor in astrocytes cultivated for a long time 5. Further investigation revealed the rare expression of CREB3L1 mRNA in the cerebral cortices from E14.5 mice, whereas high expression is recorded in the cerebral cortex at E16.5 and E18.5 during embryonic development 42. Moreover, knocking out Oasis in mice hampers astrocyte differentiation, and the cerebral cortices of Oasis-/- mice at E18.5 showed decreased quantity of glial fibrillary acidic protein (GFAP, an astrocyte marker) positive cells in contrast to these of WT mice 42, but showed increased number of neural precursor cells (NPCs) in the cerebral cortex of Oasis-/- mice. In addition, knocking out Oasis also inhibits the differentiation of cultured NPCs into astrocytes induced by leukemia inhibitory factor (LIF) and BMP2 and enhance the toxicity sensitivity of pyramidal neurons to kainic acid (KA) in the hippocampi 25. GFAP plays a critical role in astrocyte differentiation, but its expression is controlled by promoter methylation. Saito et al. proved that knockout Oasis in NPCs will attenuate the demethylation of GFAP promoter, thus contributing to the occurrence of delayed astrocyte differentiation 42. These confirm the participation of CREB3L1 in astrocyte differentiation. Glial cell missing 1 (Gcm1) is key to astrocyte differentiation. CREB3L1 can upregulate Gcm1 expression via the binding to the CRE-like element located in its promoter through forming heterodimers with CREB4. On the contrary, the N-terminal domain of CREB3 suppresses the Gcm1 expression through hampering OASIS-CREB4 heterodimer formation by competitively interacting with the CREB3L1 N-terminal domain, thus resulting in the arrest of astrocyte differentiation 25. Upon cerebral cortex injury, the elevation of CREB3L1 is detrimental to axonal regeneration. Iseki K et al. established a cryoinjured mouse brain model and found that cryoinjury mainly stimulated the CREB3L1 production in proximal reactive astrocytes accompanied by a high expression of chondroitin sulfate proteoglycans (CSPGs) composed of NG2 proteoglycan, versican, brevican, neurocan, and phosphacan core 43. Using a mouse cortical stab injury model Okuda H et al. revealed a smaller amount of CSPGs in the OASIS knockout mice compared with the wild type, this attributes to the decreased expression of chondroitin 6-O-sulfotransferase 1 (C6ST1) in Oasis-/- mice. C6ST1 serves as an enzyme implicated in sulfation of CSPGs. Overexpression of OASIS with C-terminal domain deletion in C6 glioma cells can upregulate C6ST1 expression through binding to the first intron 44. More importantly, both studies described above demonstrate that astrocytes with intact CREB3L1 suppress neurite outgrowth including NG108-15 cells and mice hippocampal neurons 43, 44. These imply that CREB3L1 can work to hamper axonal regeneration via creating a nonpermissive microenvironment. In addition, OASIS-deficient mice showed attenuated deposition of N-cadherin positive reactive astrocytes and improved functional recovery after spinal cord injury (SCI) relative to the wild type 45. These results suggest that OASIS may serve as an alternative target for the treatment of injury to CNS.

### 2.3. CREB3L1 regulates factors involved in neuroendocrine

CREB3L1 acting as a pluripotent molecule participates in the regulation of central nervous system, not only impacting on astrocyte differentiation, but also on neuroendocrine. Except astrocytes, CREB3L1 also functions in the regulation of neuroendocrine, and great progresses have been made by Mingkwan Greenwood and his colleagues. In euhydrated rats CREB3L1 expresses in magnocellular neurons (MCNs) of the supraoptic nuclei (SONs) and the paraventricular nuclei (PVNs) of the hypothalamus with a low level, whereas in the dehydrated (DH) and salt loaded (SL) rats the mRNA and protein expressions of CREB3L1 increase. Arginine vasopressin (AVP) serves as a neurohypophysial hormone participating in hydromineral homeostasis modulation. Normally it is synthesized in the MCNs of both SONs and PVNs and then transferred to and stored in the posterior pituitary. Upon the stimulation of dehydration or salt loading AVP is released into the systemic circulation to cope with the hyperosmotic stress. No matter under the EH condition or DH and SL conditions, the transcription of CREB3L1 correlates with AVP expression in SONs and PVNs, and CREB3L1 can directly modulate the transcription of AVP by binding to the CRE-like and G-box sequences located in the promoter of AVP through luciferase assay and chromatin immunoprecipitation (ChIP) 8. And cAMP can upregulate AVP levels in the hypothalamus of DH rats through elevating CREB3L1 mRNA and protein expression. However, the treatment of cAMP activator forskolin combined with glucocorticoid dexamethasone diminished the elevation of both CREB3L1 and AVP 46. More importantly, knocking down orphan nuclear receptor subfamily 4 group A member 1 (Nr4a1) in AtT20 (a mouse pituitary corticotrope tumor cell line) decreased the mRNA and protein of CEB3L1 under both the basal condition and DH condition stimulated with forskolin, further investigation revealed the transcriptional activation of Creb3l1 by Nr4a1 through binding to the NBRE site present in the promoter. This implied that the cAMP-Nr4a1-Creb3l1 signaling pathway works in AtT20 cells and the hypothalamus. However, the mRNA abundance of Creb3l1 is also affected by the methylation of Creb3l1 promoter even if the presence of forskolin stimulation 47.

Oxytocin (OXT) is another peptide hormone synthesized in MCNs, it mainly works to regulate sodium homoeostasis as a reply to hyperosmotic stimulation. CREB3L1 can act as a potential transcription factor to positively regulate the transcription of OXT in SONs and PVNs. And CAPRIN2 can binds the transcripts of OXT as well as AVP to increase their poly(A) tail lengths and mRNA abundance 48. Proprotein convertase enzyme 1 (PC1/3) is an enzyme PC1/3 mainly synthesized in neural tissues and endocrine organs and functions in processing peptide hormones, such as vasopressin and oxytocin. This enzyme is encoded by proprotein convertase subtilisin/kexin type 1 (Pcsk1). Using cell line AtT20, the supraoptic nucleus of rats, and the dehydrated rat anterior pituitary, Mingkwan Greenwood et al. demonstrated the consistent expression pattern of CREB3L1 with Pcsk1, and Creb3l1 can directly activate Pcsk1 transcription by interacting with the G-box elements located in the promoter of Pcsk1 49. Glucagon like peptide 1 receptor (GLP-1R) works as a protein implicated in glucose homeostasis through interaction with endogenous ligand glucagon like peptide 1(GLP-1). Knocking down CREB3L1 decreased the expression of GLP-1R in MCNs of the SON and PVN, further luciferase assay revealed a segment of CREB3L1 binding sequence located in the promoter of GLP-1R, implying that CREB3L1 may serve as a transcription factor for GLP-1R 50.

Except for the function described above, CREB3L1 has also been proved to be a novel ER stress sensor with similar sequence to ATF6. In the SON and PVN of rats stimulated by DH and SL CREB3L1 can be activated, which thus transcriptionally regulates the expression of DNA damage inducible transcript 3 (Ddit3) and X-Box binding protein 1 (Xbp1) 51. Protein kinase RNA-like endoplasmic reticulum kinase (PERK) acting as an ER stress sensor participates in the recovery regulation of ER function in response to stimulation. CREB3L1 comprehensively modulate PERK pathway to maintain proteostasis in magnocellular neurons, this is attributed to the direct transcription regulation of CREB3L1 on Eif2ak3, Eif2s1, Atf4, and Ddit3 52. In summary, CREB3L1 exerts a broad range of functions in modulating neuroendocrine system and may serve as a candidate target for neuroendocrine regulation.

### 2.4. CREB3L1 functions in other endocrine organs

Except neuroendocrine, CREB3L1 also functions in other secretory tissues. In the large intestine CREB3L1 expresses in the goblet cells. Disruption of CREB3L1 gene showed reduced quantity of goblet cells and decreased production of mucus. Morphologically, the goblet cells in Oasis-/- mice exhibited a few smaller secretory vesicles, and the accumulation of protein aggregation was discovered in the ER of Oasis mutant mice. Besides, Oasis-/- mice displayed a lower expression of mature goblet markers whereas a higher level of early goblet cell markers, implying the disruption of early to mature goblet cell differentiation 53. Upon the treatment of dextran sulfate sodium (DSS), Oasis-/- mice showed typical colitis in parallel with diminished body weight and elevated mortality. Further severe damage accompanied with inflammatory cell infiltration were observed in the large intestine mucosa of Oasis deficiency mice. Treatment with tauroursodeoxycholic acid can rescue the morphological and functional abnormalities in mice resulted from Oasis deficiency 54.

In thyroid cell line FRTL-5, the mRNA and protein expression of CREB3L1 can be induced by thyrotropin (TSH). Overexpression or knockdown CREB3L1 upregulate or downregulate the endogenous expression of Golgi proteins Rab1b and GM130 respectively. Transfection with full-length CREB3L1 construct or constitutively active CREB3L1 construct enlarges the Golgi volume in thyroid cells, while transfection with dominant negative CREB3L1 construct will not lead to similar internal changes. These suggest that CREB3L1 serves as a downstream molecule of TSH to upregulate the production of Golgi cargo proteins, elevate the expression of transport factors, and enlarge the volume of the Golgi apparatus to adapt to augmented cargo load, thus coping with the enhanced need of the secretory pathway related to TSH 55. Furthermore, CREB3L1 directly activates the transcription of solute carrier family 5 member 5 (Slc5a5) encoding the protein of sodium/iodide symporter (NIS) in a TSH-dependent manner. Since NIS is a thyroid protein functioned in iodide uptake. These underline the significance of CREB3L1 in the homeostasis maintenance of thyroid follicular cells, production of thyroid related proteins, and synthesis of thyroid hormone in reaction to TSH stimulation 56. In addition, the research progress regarding to the expression and function of CREB3L1 in pancreas has been well addressed by Fox RM and Andrew DJ, detailed descriptions can be seen in their review publication 26.

### 2.5. The bidirectional regulatory effects of CREB3L1 on angiogenesis

Angiogenesis plays a vital role in diverse processes of physiological and pathological conditions. The impacts of CREB3L1 on angiogenesis are emerging. Vascular endothelial growth factor A (VEGFA) acts as an important growth factor to contribute to angiogenesis. CREB3L1 can transcriptionally regulate the transcription of VEGFA in ARPE-19 cells through the CRE-like element located between -509 and -506 bp of the human VEGFA promoter 57. *In vivo* study revealed that Oasis deficiency in mice not only impaired bone formation, but also led to the decrease of blood vessel numbers. This attributes to the decline of VEGFA production. Under the hypoxic microenvironment, the amount of activated N-terminal domain of Oasis increases, which then interacts with hypoxia-inducible factor-1α (HIF-1α) coordinately to upregulate the expression of VEGFA via the binding of HIF-1α to the hypoxia response element (HRE), thus contributing to bone angiogenesis 29. However, there also exist studies showing the anti-angiogenic characteristics of CREB3L1. Enhancer of zeste 2 polycomb repressive complex 2 subunit (Ezh2) is a histone methyltransferase functioning in angiogenesis and vasculature integrity maintenance. It exerts its angiogenic function through the direct epigenetically silencing of CREB3L1 and MMP9 expression. More importantly, CREB3L1 can regulate the transcription of MMP9 in both human being and mice via binding to the sequence located at -313-+8 bp of MMP9 promoter. These suggest that upregulating CREB3L1 may hamper angiogenesis and be detrimental to vasculature integrity maintenance 30. miR-146a serves as a miRNA harboring great angiogenic potential. miR-146a overexpression improves angiogenesis and fibroblast growth factor 2 (FGF2) expression, whereas fibroblast growth factor binding protein 1 (FGFBP1) works to facilitate FGF2 activation. CREB3L1 suppresses the transcription of FGFBP1 via binding to the two CRE-like elements present at -1780 to -1777 and -868 to -865 bp of the FGFBP1 promoter in HUVECs. These results suggest that the miR-146a-CREB3L1-FGFBP1 pathway exerts a critical function during angiogenesis, while CREB3L1 serves an anti-angiogenic role 58. In breast cancer cells, overexpression of CREB3L1 in LN4D6 and MDA-MB-435 significantly diminishes cell migration and invasion. Using a rat mammary tumor model, the authors uncovered the inhibitory effects of CREB3L1 on cell survival and angiogenesis, this ascribes to the negative impacts on new vasculature formation and downregulation of FGFBP1 and Pleiotrophin 31. By summarizing the previous studies, we found that CREB3L1 exerts contradictory functions regarding to angiogenesis and vasculature integrity maintenance, these may result from the specific cell types, precise microenvironment, and specialized physiological or pathological conditions.

## 3. The pathological functions of CREB3L1

Normally, CREB3L1 mainly participates in the regulation of physiological functions. While under the conditions of aberrant expression, occurrence of somatic mutations, or generation of fusion proteins with other genes, CREB3L1 also exerts pathological functions, such as tumorigenesis and fibrosis.

### 3.1. CREB3L1 mutation implicated in osteogenesis imperfecta

Osteogenesis imperfecta (OI), also named as “brittle bone disease”, is a heritable disease usually caused by monogenic alternation related to type I collagen. The typical features of OI include decreased bone mass, reduced bone density, increased bone fragility, and frequent fractures. The estimated incidence of OI is approximately 1 to 2 per 10000 persons with no gender preference 59. Originally, the Sillence Classification of OI including fourth subtypes was determined according to the clinical and radiological characteristics of patients 60. Up to now, the classification of OI has been revised several times. The current version of OI classification harbors 18 subtypes, the detailed information can be seen in the publication written by Darran Mc Donald et al. 61 Presently, OI is deemed to be a disorder with genetic heterogeneity. However, nearly 90% of the OI cases resulted from the causative genetic mutations found in either COL1A1 or COL1A2 gene 62. The remaining OI cases are caused by mutations occurred in other genes. CREB3L1 represents one of them. In mice CREB3L1 deficiency leads to severe osteopenia showing decreased bone density. In human OI resulted from CREB3L1 mutations is allocated into subtype XVI and functional group E. The phenotypes of OI patients affected by CREB3L1 genetic alternations vary considerably. Symoens S et al. firstly reported the cases of human OI associated with CREB3L1 abnormality happened in a Turkish family with two affected individuals 63. One case showed several fractures in utero and small stature corresponding to his gestational age. During the developmental stage after birth, he developed fractures in extremities and tubular bones. Beaded ribs and callus formation were also observed. As a result of various causes, the boy died at 9 months. The other affected one was a male foetus from medical terminated gestation who exhibited thin ribs and diverse fractures during autopsy. PCR amplification and array CGH analysis confirmed the absent expression of CREB3L1 and a homozygous whole gene deletion of CREB3L1. Whereas their parents and healthy family member showed heterozygous deletion of CREB3L1. These revealed the possible relationship between OI and CREB3L1 dysfunction 63. Keller RB et al. reported a family suffered from prenatal and perinatal lethal OI, exome sequencing on the affected and unaffected individuals revealed a 3 bp inframe deletion (c.934_936delAAG [p. Lys312del]) (in exon 7) in CREB3L1 gene. The affected individuals harboring a homozygous variant of CREB3L1 showed typical features of OI type II, while the family members containing a heterozygous mutation exhibited a mild phenotype of OI 64. Lindahl K et al. showed a Somali boy affected by severe OI, who exhibited abnormal phenotype and hypermineralized bone matrix. These resulted from the generation of a homozygous stop codon variant in NM_052854.2 transcript of CREB3L1 (c.1284C > A; p. Tyr428*) (exon 11) 65. Guillemyn B et al. presented a Turkish family suffered from terminated pregnancy resulting from OI and identified the mutated CREB3L1 as the causative gene. A homozygous missense mutation [c.911C>T p. (Ala304Val)] was detected in exon 7 of patient CREB3L1 gene. The protein encoded by the mutated transcript of CREB3L1 hindered expressions of genes encoded Col1 and COPII vesicle proteins 66. Cayami FK et al. described a family with adult OI resulting from a novel homozygous genetic alternation (c.1365del) in exon 11 of causative gene CREB3L1, which leads to the production of frameshift in NM_052854.3 (p. Pro458Argfs*25). Three individuals with homozygous variant were born healthy but affected in adulthood by OI, while their parents and a brother with heterozygous variant of CREB3L1 were not clinically affected and showed no typical phenotypes of OI 67. Andersson K et al. reported a 12-year-old male OI subtype III patient with oligodontia and severe malocclusion. A homozygous nonsense mutation (c.1284C > A, p. Tyr428*) was detected in CREB3L1. The variant generates a stop codon in transcript NM_052854.3 at position 428 and leads to the early termination of protein translation 68. Sarah E Lindsay et al. showed an 18-year-old female OI patient with frequent fractures. Both lower and upper extremities shortening and bowing and severe fibrous dysplasia were found. Genetic analysis revealed a c.119C>T (p. Ser40Leu) mutation in exon 1 of Interferon-induced transmembrane protein 5 (IFITM5), and a c.676C>A (Pro226Thr) mutation was discovered in exon 5 of CREB3L1. Both mutations in these two genes are heterogeneous. The authors inferred that the phenotypic changes of patients should ascribe to the genetic alternation of IFITM5, for the typical clinical and radiographic characteristics of OI subtype V are absent. This is consistent with several previous reports resulted from the heterozygous mutation in IFITM5. The mutation found in CREB3L1 was novel and predicted to be benign using software modeling 69. David P DeMasters et al. reported a 31-year-old female patient of OI subtype I experiencing polycystic ovary syndrome, mild (right ear) and moderate (left ear), and migraines, gray-blue sclera and multiple prior fractures were also observed. These are attributed to the heterozygous mutation of CREB3L1 gene, unfortunately the detailed genetic change and amino acid substitution were not shown 70. Detailed information about the OI cases due to mutation of CREB3L1 is listed in Table 1.

### 3.2. CREB3L1 in tumorigenesis

#### 3.2.1. CREB3L1 in low grade fibromyxoid sarcoma (LGFMS) and sclerosing epithelioid fibrosarcoma (SEF)

Low grade fibromyxoid sarcoma (LGFMS) is a malignant soft tissue tumor firstly described by Evans in 1987 71. Its clinical features, ultrastructural characteristics, genetic variations, pathologic changes, and immunohistochemical phenotype have been clearly depicted by Mohamed M et al. 72 Increasing evidence demonstrated that both CREB3L1 and CREB3L2 contribute to LGFMS. Vast majority of the LGFMS harbor a balanced translocation, t (7;16) (q34; p11), which lead to the generation of Fused in sarcoma (FUS)-CREB3L2 variant. There also exist a small number of LGFMS cases exhibiting a variant FUS-CREB3L1 fusion caused by t (11;16) (p11; p11). Besides, EWSR1-CREB3L1 fusion was also reported in LGFMS 73. However, the points and base sequences of FUS, CREB3L1, and CREB3L2 involved in fusion are diverse 74. Mertens F et al. firstly reported a fusion of FUS exon 9 with CREB3L1 exon 5 in LGFMS 75. Guillou L et al. showed three LGFMS cases harboring FUS exon 6-CREB3L1 exon 5 and FUS exon 6-CREB3L1 exon 5 (entire) variants found in thorax and knee 74. Hisaoka M et al. revealed a LGFMS with a fusion gene resulting from the conjunction of FUS exon 6 and CREB3L2 exon 5 76. Odem JL et al. recorded a LGFMS case showing a loss of 11p11.2p15.5 accompanied by a gain of 16p11.2p13.3, implying the production of 5′-FUS/3′-CREB3L1 gene involved in tumor formation 77.

Sclerosing epithelioid fibrosarcoma (SEF) represents a rare fibroblastic tumor firstly reported by Meis Kindblom et al. The affected individuals are of different ages and distinct nationalities 72, 78. It is histologically comprised of nests and cords of epithelioid cells containing clear or eosinophilic cytoplasm located in the sclerotic stroma and genetically characterized by the finding of EWS RNA Binding Protein 1 (EWSR1) rearrangements, presence of EWSR1-CREB3L1 variant, or occurrence of EWSR1-CREB3L2 gene fusion 72. The EWSR1-CREB3L1 variant only appeared in a minority of SEF cases. Arbajian E et al. reported 4 cases of pure SEF with EWSR1-CREB3L1 variants, among them cases 2-4 showing in-frame fusions of EWSR1 exons 11, 8, and 7 to CREB3L1 exon 6 respectively 79. Stockman DL et al. reported a fusion gene between intron 11 of EWSR1 (22q12.2) and intron 5 of CREB3L1 (11p11.2) in a 43-year-old male SEF patient 80. There also exist studies showing pure SEF with an in-frame fusion of PAX5 exon 2 with CREB3L1 exon 6 81 and FUS-CREB3L1 chimeric transcript 82. These affected locations involve chest wall, groin, pelvis, shoulder, omentum, mesentery, peritoneum, serosal surfaces of bowel, spine, bone, femur, thigh, abdomen, forehead, pleural, retroperitoneum, sacrum, lung, stomach, lesser curvature, chest wall, left anterior, paranasal sinuses, orbit and frontal bone, retroperitoneum, and jaw 72, 79, 80, 82-90. Except for the effects of CREB3L1 mutations on sarcoma formation, the expression of CREB3L1 can also serve as a marker for the efficacy and survival prediction of doxorubicin based palliative chemotherapy for patients with advanced soft tissue sarcoma 91.

#### 3.2.2. CREB3L1 in glioma

Except for the cultivated astrocytes, CREB3L1 expression in brain glioma tissues was also recorded. Owing to the high methylation status of CREB3L1 promoter, a proportion of tumors displayed low CREB3L1 expression, target removal of promoter hypermethylation hampers glioblastomas formation in nude mice. Downregulated CREB3L1 expression and functionally mutated CREB3L1 facilitate glioblastoma proliferation, correspondingly recovery of CREB3L1 hinders glioblastoma formation 92. Interestingly, in brain glioma the expression of CREB3L1 negatively correlates with PTN levels, absence of CREB3L1 accompanied by the presence of PTN implied a short survival time and poor prognosis for brain glioma patients 93.

In astrocytes, CREB3L1 arrests cells in G2/M phage via transcriptionally activating P21 expression 92, while in glioma cell lines, ER stress inducing compounds including tunicamycin or thapsigargin induce the expression of CREB3L1, knock-down of CREB3L1 leads to the attenuation of UPR proteins GRP78 and GRP94 and reduced extracellular matrix protein expression except COL1. And CREB3L1 reduction contributes to cell morphology change and diminishes cell migration 7.

#### 3.2.3. CREB3L1 in breast cancer

Breast cancer represents the most commonly cancer occurred in women, which occupies 11.7% of cancers diagnosed in female 94. Normally, low and medium grade breast tumors show increased CREB3L1 expression, while in advanced and metastatic breast cancers the expression of CREB3L1 was suppressed attributing to the preferential methylation of CpG island. Decreased expression of CREB3L1 is related to high tumor grade and implies a bad prognosis and short survival time for patients with tumors positive for estrogen receptor (ER) and negative for human epidermal growth factor receptor 2 (HER2) (luminal A) and triple negative breast cancer (TNBC) 95, 96. PERK signaling serves as a critical pathway functioned in breast cancer invasion and metastasis. CREB3L1 can work to facilitate breast cancer metastasis through mediating the prometastatic signaling of PERK, especially for TNBCs. Suppression of CREB3L1 is conducive to hampering cancer cell invasion and metastasis 97. Up to now, chemotherapy still serves as the main treatment for metastatic TNBC. Doxorubicin and paclitaxel representing two of the chemotherapeutics have been extensively studied, emerged evidence showed that doxorubicin suppresses tumor cell proliferation via activating CREB3L1 98, while paclitaxel works to elevate ceramide generation to facilitate the translocation of CREB3L1 from ER to Golgi complex 95. Deletion of CREB3L1 in MDAMB231 cell line suppresses GRP78 expression and abolishes the inhibitory function of CREB3L1 on metastasis 99. However, most of the TNBCs showed downregulated CREB3L1 expression, its effectiveness and sensitivity to chemotherapy are limited. Combination treatment with homoharringtonine and paclitaxel exhibits synergistic cytotoxic effects on TNBC cells accompanied by decreased toxic side effects compared with separate curing 100.

#### 3.2.4. CREB3L1 in thyroid cancer

Thyroid cancer represents the most common endocrine cancer with 586,202 new cases in 2020 all over the world 94. It is comprised of various histological types, papillary thyroid cancer (PTC) and anaplastic thyroid cancer (ATC) are two of them. PTC stands for the well differentiated type with a good prognosis, while ATC represents an undifferentiated type displaying aggressive phenotype and accounts for 40% of the thyroid cancer deaths 101. Among the tissues of normal thyroid (NT), PTC, and ATC, the mRNA and protein expressions of CREB3L1 in PTC and ATC are higher than NT, whereas ATC shows the highest expression. The amounts of CREB3L1 in cells of NT, PTC, and ATC present similar pattern to tissues. Bioinformatics analysis revealed distinct regulatory networks between PTC and ATC, and the high expression of CREB3L1 is positively correlated with tumor stage and negatively correlated with overall survival (OS) 101-103. Using single cell sequencing, Luo H et al. found that CREB3L1 is expressed in most of the ATC cells and gradually enriched along with the follicular-aPTC-ATC trajectory. More importantly, the target genes of CREB3L1 are inclined to synchronizing with ATC progression and implicated in EMT and mTORC1 pathways. And the positive staining for CREB3L1 was associated with PTC relapse. These implied that CREB3L1 is critical for thyroid cancer cell fate determination and ATC progression 104. Further, study from Pan Z et al. demonstrates that lots of ATC cells harbor the cancer associated fibroblasts (CAFs) phenotype via co-expression of CREB3L1 and fibroblast activation protein alpha (FAP), knocking down CREB3L1 in ATC cells significantly attenuates the expressions of collagens of distinct types. COL5A1 acts as one of them and serves as a direct downstream target of CREB3L1. CREB3L1 promotes ATC metastasis through upregulating COL5A1 expression. Further, CREB3L1 can remodel the microenvironment of ATC by mediating cell interaction between ATC cells and CAFs, especially for α‑SMA positive CAFs. More importantly, karyopherin subunit alpha 2 (KPNA2) can interact with CREB3L1 to facilitate the nuclear translocation of CREB3L1, which then augments IL‑1α synthesis to promote α‑SMA positive CAFs differentiation. In addition, the authors also confirmed that CREB3L1 contributes to the malignant phenotype maintenance of ATC via mediating the metastasis of ATC cell derived xenograft in zebrafish and nude mice 102.

#### 3.2.5. CREB3L1 in other cancers

Except for these described above, CREB3L1 is also implicated in some other cancers. The expression of CREB3L1 is downregulated in bladder cancer tissues due to promoter hypermethylation, its protein deletion is often found in the nuclei of bladder cancer cells with aggressive phenotype. Overexpression of CREB3L1 in bladder cell lines without endogenous CREB3L1 inhibits cell migration and colony growth. And the CREB3L1-HTRA3 axis implicated in bladder cancer development 105. The expression of CREB3L1 in prostatic cancer cell line LNCaP is moderate, knocking down CREB3L1 attenuates p21 expression. Androgen induced bZIP (AIbZIP) can suppress p21 expression through inhibiting S2P-mediated proteolysis of CREB3L1 by binding to CREB3L1 at the Golgi apparatus 106. And bioinformatics analysis using RegNet Driver revealed CREB3L1 as a novel transcription factor functioned in prostatic cancer 107.

#### 3.3 CREB3L1 in fibrosis

Fibrosis often occurs after tissue or organ injury characterized by the accumulation of extracellular matrix (ECM). Moderate fibrosis is beneficial for wound healing and restoring tissue structure and function. However, severe fibrosis results in excessive ECM deposition, which leads to the destruction of tissue architecture and/or organ dysfunction, thus endangering human normal life and health. During the initiation, development, and progress of fibrosis, TGF-β/SMAD signaling pathways play an important role and function in promoting tissue fibrosis 108. CREB3L1 acting as a downstream pleiotropic factor of TGF-β/SMAD signaling pathway also functions in fibrogenesis. In response to the acute stimulation of TGF-β, TGF-β receptors TGFβRI and TGFβRII are activated and form a heterodimerization to phosphorylate Smad2/3, which then interact with Smad4 to induce target genes expression. Upon the chronic stimulation of TGF-β, phosphorylation of ERKs initiates, then the phosphorylated ERKs suppresses transmembrane 4l six family member 20 (TM4SF20). The decreased TM4SF20 facilitates the activation of RIP for CREB3L1 to release the N-terminal domain, which then forms a complex with Smad4 and translocate to the nuclei to activate the transcription of downstream target genes 2, 109, thus contributing to tissue fibrosis.

Renal fibrosis often occurs in patients with kidney diseases and links to dysfunction of affected patients. Yamamoto A et al. first revealed the function of CREB3L1 in renal fibrosis. They demonstrated the elevated expression of CREB3L1 in kidneys from human chronic kidney disease (CKD) patients and fibrosis mouse model using the publicly available database Nephroseq, and a large number of cells display nuclei staining for CREB3L1 in the tubulointerstitium, whereas in the tubular epithelial cells of normal human kidney CREB3L1 protein mainly shows cytoplasmic localization. Using a mouse unilateral ureteral obstruction (UUO) model of kidney fibrosis, they uncovered a time dependent increase of CREB3L1 mRNA and its active form of protein. In addition, the mRNA levels of CREB3L1 and α-SMA were upregulated in renal interstitial fibrotic tissues resulting from folic acid treatment or ischemia and reperfusion (I/R) injury. In mouse and human fibrotic renal tissues lots of CREB3L1 and α-SMA double positive myofibroblasts were observed. TGF-β1 treatment stimulates the production of CREB3L1 of both full length and active form coincident with enhanced α-SMA in renal fibroblasts. And CREB3L1 is required for TGF-β1 stimulated myofibroblast migration and proliferation. Deletion of CREB3L1 improves kidney fibrosis. The fibrogenic function of CREB3L1 in kidneys relies on the transcriptional regulation of bone marrow stromal antigen 2 (Bst2) 110. Furthermore, study on transgenic mice with the overexpressed active form of CREB3L1 in podocyte shows tubular injury and tubulointerstitial fibrosis 111. Liver fibrosis is another common fibrogenic disease resulted from nonalcoholic fatty liver disease (NAFLD) or nonalcoholic steatohepatitis (NASH). The activated hepatic stellate cells (HSCs) are the main cell source facilitating liver fibrosis. The results of human NASH tissue sequencing reveal increased CREB3L1 expression accompanied by the evolution of healthy tissues to NASH with fibrosis. Further study shows that CREB3L1 can act as a major regulator to contribute to HSC activation and liver fibrosis through regulating liver fibrosis related genes 112. Besides, studies also implicate the potential involvement of CREB3L1 in cutaneous fibrosis disease. mRNA sequencing uncovered elevated transcript of CREB3L1 in keloids, and the protein predominates in fibroblasts 9. While in hypertrophic scars more FB-1 cells (a subtype of fibroblasts) express CREB3L1, and the transcriptional activities of CREB3L1 was higher in FB-1 cells in hypertrophic scar than that in normal skin, which is then implicated in myofibroblast to work as a transcription factor 112. Although existing studies have revealed the role of CREB3L1 in fibrosis, the detailed mechanism remains unknown and requires further investigation.

## 4. Conclusions

This review uncovers the expression characteristics of CREB3L1 in different tissues and reveals its upstream and downstream regulatory network. Existing evidence has confirmed its physiological roles in bone and tooth development, functions in central nervous system, contributions to neuroendocrine and secretory cell differentiation, and modulations of angiogenesis through regulating the expressions of distinct genes.

**Figure 3:**
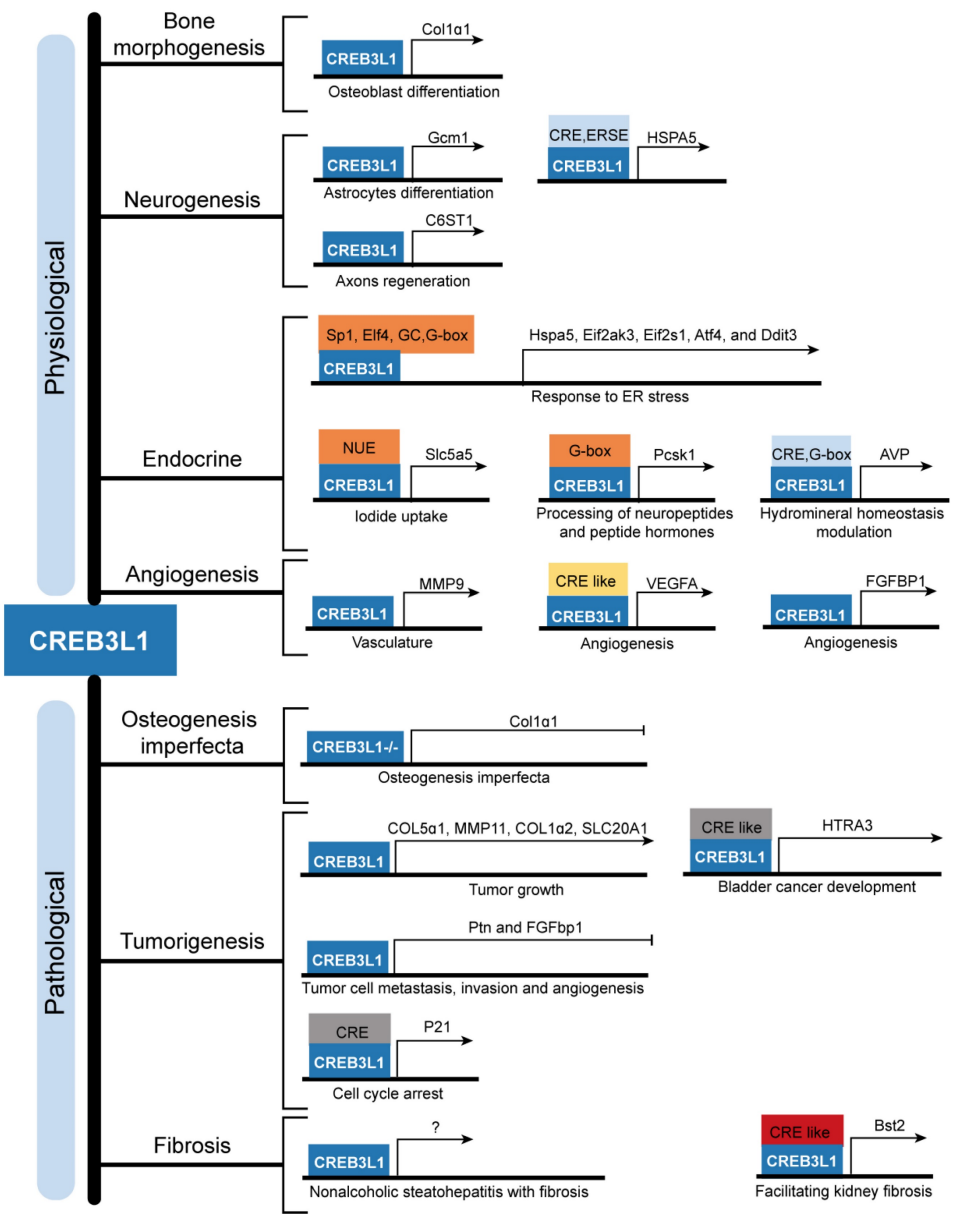
** The downstream targets of CREB3L1 and their corresponding physiological and pathological functions.** CREB3L1 transcriptionally regulates the expressions of downstream target genes through binding to the cAMP responsive element (CRE), CRE-like sequence, ER stress response element (ERSE), Sp1 TF binding sequence, Elf4 TF binding sequence, GC rich sequence, G-box sequence, G-box like sequence, and CRE element inside the NIS upstream enhancer (NUE) present in the promoters or intron to modulate bone development, neurogenesis, endocrine, angiogenesis, OI, tumorigenesis, and fibrosis.

Further emerging evidences are highlighting the pathological functions of abnormally expressed CREB3L1 in the occurrence, progress, and prediction of patients with glioma, breast cancer, and thyroid cancer. Besides, the relationship between individual somatic mutations in CREB3L1 and OI has been revealed. And FUS-CREB3L1 fusion gene and EWSR1-CREB3L1 variants are implicated in LGFMS and SEF respectively. In addition, CREB3L1 is involved in diverse fibrotic diseases, including renal fibrosis, liver fibrosis, hypertrophic scar, and keloids (Figure 3). However, the detailed mechanisms of how CREB3L1 promotes the pathogenesis of the aforementioned diseases are largely undefined, more researches are urgently required. Nevertheless, multiple involvement of CREB3L1 suggests that it may serve as a candidate target for the treatment of cancers and fibrosis. Minimizing the production of active CREB3L1 by inhibiting RIP or targeting downregulation of CREB3L1 mRNA expression using gene intervention methods are the potential feasible strategies. In conclusion, this review deepens our understanding on CREB3L1 both physiologically and pathologically and lays a foundation for further study on CREB3L1 and its functions in clinical practice.

## Figures and Tables

**Table 1:** CREB3L1 mutations that cause OI.

Age/sex	Mutation	Amino acid change	Status	Sequencing methods	Year	References
9 months/male; 19-week-old foetus/male	Whole gene deletion	/	Homozygous	PCR and Array CGH	2013	63
11 years/male	c.1284C > A	p. Tyr428*	Homozygous	Next generation sequencing and Sanger sequencing	2018	65
18-week-old embryo (three affected individuals)	c.934_936del AAG	p. Lys312del	Homozygous	Exome and Sanger sequencing	2018	64
Three patients less than 4 years/male	c.1365del	p. Pro458Argfs*25	Homozygous	Next generation Sequencing and Sanger sequencing	2019	67
19-week-old foetus/female	c.911C>T	p. Ala304Val	Homozygous	Panel sequencing and Sanger sequencing	2019	66
12 years/male	c.1284C > A	p. Tyr428*	Homozygous	Whole genome sequencing and Sanger sequencing	2020	68
31 years/female	/	/	Heterozygous	/	2022	70

## Abbreviations

ATC: Anaplastic thyroid cancer
AODs: Apical odontoblasts
AVP: Arginine vasopressin
BBF2H7: BBF2 human homolog on chromosome 7
BMP2: Bone morphogenetic protein 2
CREB3: cAMP responsive element binding protein 3
C6ST1: Chondroitin 6-O-sulfotransferase 1
CSPGs: Chondroitin sulfate proteoglycans
CKD: Chronic kidney disease
DH: Dehydrated
ER: Endoplasmic reticulum
FGF2: Fibroblast growth factor 2
Gcm1: Glial cell missing 1
GFAP: Glial fibrillary acidic protein
GLP-1R: Glucagon like peptide 1 receptor
Hsp70: Heat shock protein family A
HSCs: Hepatic stellate cells
HIF-1α: Hypoxia-inducible factor-1α
LIF: Leukemia inhibitory factor
LGFMS: Low grade fibromyxoid sarcoma
MCNs: Magnocellular neurons
NPCs: Neural precursor cells
NAFLD: Nonalcoholic fatty liver disease
Nr4a1: Nuclear receptor subfamily 4 group A member 1
OI: Osteogenesis imperfecta
OXT: Oxytocin
PTC: Papillary thyroid cancer
PVNs: Paraventricular nuclei
PDLSCs: Periodontal ligament stem cells
PC1/3: Proprotein convertase enzyme 1
RIP: Regulated intramembrane proteolysis
Runx2: RUNX family transcription factor 2
SEF: Sclerosing epithelioid fibrosarcoma
SCI: Spinal cord injury
SONs: Supraoptic nuclei
TSH: Thyrotropin
NT: Tissues of normal thyroid
TGF-β: Transforming growth factor-β
Col1: Type I collagen
UUO: Unilateral ureteral obstruction
VEGFA: Vascular endothelial growth factor A
Xbp1: X-Box binding protein 1
